# Human Immunodeficiency Virus Preexposure Prophylaxis Knowledge, Attitudes and Perceptions of Sexual Health Risk in an Age of Sexually Transmitted Infection Antimicrobial Resistance

**DOI:** 10.1097/OLQ.0000000000001384

**Published:** 2021-02-03

**Authors:** Ava Lorenc, Jane Nicholls, Joanna May Kesten, Louis Macgregor, Nathan Speare, Lindsey Harryman, Katy M.E. Turner, Patrick Horner, Jeremy Horwood

**Affiliations:** From the ∗ National Institute for Health Research Applied Research Collaboration West (NIHR ARC West), University of Bristol, University Hospitals Bristol NHS Foundation Trust; † Cardiff and Vale Sexual Health Service, Cardiff; ‡ Population Health Sciences, University of Bristol, Oakfield House, Oakfield Grove, Bristol; § Unity Sexual Health, University Hospitals Bristol and Weston NHS Foundation Trust; ¶ Bristol Veterinary School, University of Bristol, Langford, United Kingdom

## Abstract

**Background:**

Human immunodeficiency virus (HIV) preexposure prophylaxis (PrEP) has helped reduce new HIV infections. However, bacterial sexually transmitted infections (STIs) have increased among PrEP users. We examined PrEP knowledge, access, and risk perceptions in an age of antimicrobial resistance (AMR).

**Methods:**

An online anonymous survey was distributed to all cisgender men/transpersons who have sex with men attending a sexual health clinic in Bristol, United Kingdom (October 2018 to November 2019). Interviews with a sample identified at increased risk of HIV were analyzed thematically and integrated with survey data.

**Results:**

Five hundred and seventy-eight (95%) of 617 cisgender men/transpersons who have sex with men survey respondents were HIV-negative/unknown, of these, 202 (34.9%) had ever used PrEP. Interviewees (n = 24) reported widespread awareness of and enthusiasm for PrEP. Among nonusers, 39% (146/376) were unaware how to access PrEP, and 27% (103/376) could not access PrEP through the national “impact” trial of whom 79% (81/103) were eligible. The PrEP was described as “life-changing,” but expense was the main barrier to use. Sixty-two percent (358/578) of HIV-negative/unknown respondents on PrEP were more likely to have condomless anal intercourse with someone they thought was HIV-negative. Interviewees used PrEP with other risk-reduction strategies. Sexually transmitted infections were seen as “curable” and AMR rarely influenced risk perception or sexual decision making.

**Conclusions:**

The PrEP awareness was high, but purchase cost limited access. PrEP may increase condomless anal intercourse, but interviewees used PrEP as one of many risk-reduction tools. Reduced fear of HIV transmission and testing was highly valued. Sexually transmitted infection AMR was not seen as an immediate threat and did not influence risk perception or sexual decision making.

## Abstract 2

A mixed methods study of cisgender men/transpersons who have sex with men sexual health clinic attendees found that preexposure prophylaxis reduced anxiety and may increase likelihood of condomless anal intercourse, but risk of sexually transmitted infection antimicrobial resistance was not seen as an immediate threat.

Supplemental digital content is available in the text.

Human immunodeficiency virus (HIV) preexposure prophylaxis (PrEP) is the use of HIV treatment medications by HIV-negative people to prevent them becoming infected. Since 2015 PrEP has been established as an effective HIV intervention among cisgender men who have sex with men (MSM) and transpersons who have sex with men (TPSM), with clinical trials showing between 86% and 97% efficacy in reducing HIV incidence.^1^ Studies have shown reduced fear and anxiety around HIV in PrEP users.^2^ The recent impressive reduction of new HIV infections among MSM in London (by 40% between 2009 and 2018)^3,4^ is thought to be due to prevention measures, including PrEP. However, this reduction has not been reproduced as significantly in other areas of the United Kingdom.^5^ United Kingdom access to PrEP is variable and location dependent, especially outside London,^6^ with 1 in 5 individuals unsuccessful in obtaining PrEP.^7^ At the time this study was conducted, PrEP was freely available through the NHS in Wales and Scotland but in England only as part of a research trial (the “Impact” trial) to those meeting all specific eligibility enrolment criteria. It is planned to be rolled out free of charge across the United Kingdom over 2020 to 2021.^8^

The PrEP is only recommended for individuals at increased risk of HIV. Eligibility for MSM in England (on the Impact trial) is based on the following: ongoing condomless anal intercourse (CAI) or; a regular sexual partner with HIV with a detectable viral load; or other factors posing similar HIV-risk, with similar eligibility criteria in Scotland and Wales.^9^ Personal purchase of PrEP online is common^7,9^ but only around half of private PrEP users reported regular STI testing or renal function monitoring.^7^

Sexually transmitted infection incidence is increasing,^10^ with high rates of CAI and STIs among PrEP users in the United Kingdom and globally.^11–13^ Previous research suggests that PrEP users may have a heightened awareness of their health and well-being^13^ and increased engagement in health care.^14^ Around half of United Kingdom PrEP users report an STI diagnosis each year.^7^^,13^ At the same time, both gonorrhea and gonococcal antimicrobial resistance (AMR) are increasing in the United Kingdom,^15,16^ including cases of extensively drug resistant *Neisseria gonorrhea*^15^ and is a major international public health concern.^17^ An understanding of PrEP users' risk perception is vital to understand sexual decision making and attitudes to STIs in an age of AMR.^10^^,13^

We explored knowledge about and access to PrEP, PrEP's impact on sexual decision making and risk perception of acquisition of HIV and STIs, including those resistant to treatment, among MSM/TPSM attending a United Kingdom sexual health clinic outside London during the Impact trial.

## MATERIALS AND METHODS

### Design

Online survey and qualitative interviews.

### Setting

A sexual health center in Bristol, United Kingdom (population 450,000), a site for the Impact trial (www.prepimpacttrial.org.uk).

### Participants

All MSM/TPSM clinic attendees from October 2018 to November 2019 were sent the survey. Survey respondents were asked to indicate willingness to be interviewed.

### Data Collection

#### Survey

An anonymous self-completion online survey was developed on Research Electronic Data Capture and piloted through 4 rounds of cognitive interviewing with a convenience sample of 10 MSM to evaluate and refine face validity. The final survey contained questions on sociodemographic characteristics, sexual health service use, sexual behavior in previous 3 months, and views and experience of PrEP.

A weblink to the survey was sent by smartphone text to clinic attendees after clinic attendance.

#### Interviews

Qualitative interviews explored views and experiences of PrEP, using an open-ended topic guide (Supplemental Digital Content 1, http://links.lww.com/OLQ/A625). From PrEP eligible survey respondents with an elevated HIV risk score (see below), interviewees were purposively sampled regarding age, gender, ethnicity, socioeconomic status (estimated using the Index of Multiple Deprivation Decile), and PrEP usage. A.L. and J.K. invited potential interviewees to participate by text message, telephone, or email and conducted 30- to 60-minute interviews by phone, or face-to-face between April and November 2019. Informed consent (written for face-to-face, verbal for phone interviews) was obtained before interviews. Interviewees were offered a £20 high street voucher. The sample size was driven by the concept of “information power,”^18^ with continuous assessment of sample information regarding study objectives.

### Analysis

#### Survey

Descriptive analyses were performed on the survey responses using STATA v15. Eligibility for inclusion within analysis required the following: consent to participate, MSM/TPSM self-identification and complete responses to all questions regarding HIV-status and sexual decision making concerning PrEP use. Any sections in which respondents did not answer (resulting in missing data) were excluded from the analysis.

Participants were deemed eligible for PrEP if reporting CAI in the past 3 months. The PrEP eligible population were subdivided into “elevated” and “lower” HIV risk groups based on the presence of 1 or more risk factors associated with greater odds of HIV infection. Self-identified risk factors (past 12 months) included the following: diagnosis of rectal chlamydia, rectal gonorrhea or syphilis, use of postexposure prophylaxis, 5 or more partners or participation in chemsex (sexual intercourse under effects of mephedrone, crystal methamphetamine or gamma hydroxybutyrate/gamma butyrolactone).

#### Interview

Interviews were digitally audiorecorded, transcribed verbatim, anonymized, imported into NVivo 10 (QSR International) and analyzed thematically by A.L.^19^ A subset of transcripts was independently analyzed by J.H. to aid code refinement and maximize rigor. Codes were built into broader categories and themes discussed by the multidisciplinary research team to ensure credibility.

The integration of qualitative and quantitative data used the established “following a thread” technique^20^—tracing key themes using all data sets.

#### Patient and Public Involvement

Two patient and public involvement meetings developed the study procedures and documentation with 2 MSM sexual health clinic users. These meetings informed survey/interview recruitment and provided feedback on the participant information sheet, interview topic guide, and the survey. The electronic survey was further tested to make sure it was usable (on a phone) and understandable.

#### Ethical Approval

Ethics approval was obtained from the NHS Health Research Authority, National Research Ethics Service Committee South West—Frenchay Research Ethics Committee (18/SW/0142).

## RESULTS

### Participants

The survey was distributed to 1975 TPSM/MSM; 1140 gave consent and provided responses. We retained an analytic sample of 617 after eliminating those who did not provide complete responses to all questions in sections regarding HIV status and sexual decision making (see above).

Although we were unable to collect information from nonparticipants, the 617 survey respondents were largely similar to all MSM/TPSM clinic attendees over the same time period (Table 1). However, survey respondents were more ethnically diverse. Interviews were conducted with 24 survey respondents and included 23 MSM and 1 nonbinary individual. Survey respondents and interviewees had statistically similar indices of multiple deprivation range to clinic attendees.

**TABLE 1:** Comparison of Demographic Characteristics of MSM/TPSM Survey Participants, Interviewees and MSM/TPSM Clinic Attendees October 31, 2018, to November 15, 2019

Demographics	No. Survey Respondents (%)		No. Interview Respondents (%)		No. Clinic Attendees* (%)		*n* (df)^†^ *χ*^2^*P*
Age, y	617		24		1934		2596 (3)
<30	198	32%	13	54%	890	46%	49
30–49	293	48%	8	33%	809	42%	<0.0001
>50	126	20%	3	13%	235	12%	
Ethnicity	617		24		1856		2515 (7)
White British	413	67%	14	58%	1385	75%	36
White other	114	19%	6	25%	286	15%	<0.0001
Mixed/multiple ethnic groups	29	5%	2	8%	42	2%	
Asian/Asian British	34	6%	1	4%	67	4%	
Black/African/Caribbean/Black British	13	2%	1	4%	16	1%	
Prefer not to say	9	2%	0	0%	45	2%	
Other	5	1%	0	0%	15	1%	
Highest qualification	616		24		—		—
No educational qualifications	10	2%	0	0%	—	—	—
GCSEs or equivalent	62	10%	4	17%	—	—	—
A-levels or equivalent	81	13%	6	25%	—	—	
BTEC/NVQ/diploma or equivalent	80	13%	1	4%	—	—	
University degree or higher	378	61%	13	54%	—	—	
Other	5	1%	0	0%	—	—	
IMD**^†^**	398		24		1830		2256 (5)
Quintile 1 (most deprived)	64	16%	4	17%	359	20%	8
Quintile 2	89	22%	6	25%	435	24%	0.0770
Quintile 3	99	25%	6	25%	376	20%	
Quintile 4	72	18%	6	25%	377	20%	
Quintile 5 (least deprived)	74	19%	2	8%	283	16%	
Sexuality	617		24		—		—
Sex with men	499	81%	22	92%	—	—	—
Sex with men and women	64	10%	1	4%	—	—	—
Other sexual preference	54	9%	1	4%	—	—	
Gender	617		24		—		—
Cis male	599	97%	23	96%	—	—	—
Transgender	5	1%	0	0%	—	—	—
Nonbinary/other	13	2%	1	4%	—	—	
HIV status	617		24		—		—
Unaware of their HIV status	26	4%	0	0%	—	—	—
HIV-positive	39	6%	0	0%	—	—	—
HIV-negative	552	90%	24	100%	—	—	
PrEP use (in HIV-negative MSM)	578		24		—		—
PrEP eligible (total)	402	70%	24	100%	—	—	—
Has never used PrEP and is ineligible	155	27%	0	0%	—	—	—
Has never used PrEP but is eligible	221	38%	14	58%	—	—	
Currently using PrEP and is ineligible	12	2%	0	0.0%	—	—	
Currently using PrEP and is eligible	162	28%	8	33%	—	—	
Previously used PrEP and is ineligible	9	2%	0	0%	—	—	
Previously used PrEP and is eligible	19	3%	2	9%	—	—	

*Multiple visits may be associated with each clinic identification number; we use demographic information relating to the most recent visit between October 31, 2018, and November 15, 2019.

^†^*χ*^2^ test of goodness of fit, expressed with degrees of freedom (*df*) and *P* value between survey and clinical data sets.

^‡^IMD is an overall measure of the relative deprivation based on geographical area of residence (specifically lower layer super output area).

IMD, Index of multiple deprivation.

### PrEP Knowledge, Use, and Access

All HIV-negative/status unsure survey respondents had heard of PrEP. Of survey respondents who were HIV-negative/status unsure, 202 (35%) of 578 had ever taken PrEP and 174 (86%) of 202 were taking it currently. Most PrEP users obtained PrEP from the Impact trial (108/202; 54%) or online (86/202; 43%), of which 69 (80%) of 86 used www.Iwantprepnow.co.uk recommended sites. Only 72 (36%) 202 had discussed their PrEP use with their GP.

Nine interviewees were currently taking PrEP, 2 were about to start taking it, and 4 were on the Impact trial waiting list. Among interviewees, PrEP use and knowledge was perceived as embedded in the MSM community. The most common interviewee gaps in PrEP knowledge regarded adverse effects, dosing regimens, cost, and where to buy it. Those on the Impact trial felt “ridiculously lucky.” PrEP was described as “gift from the Gods,” “life-changing” and the potential to reduce HIV prevalence and stigma was applauded.

“I think it is [PrEP] amazing, yeah. The idea behind it, the fact that if everyone went on PrEP, within a couple of years we could eradicate HIV in this country” (Tim, never used PrEP)

Reasons for taking PrEP were as extra protection from HIV, to avoid condoms (to increase sexual performance or pleasure), or as a “duty” to reduce HIV prevalence.

“I can't get, really have a hard-on [with condoms]…[With PrEP] it's like, ‘oh wow and I can have sort of protection that way and I don't have to use the condoms’” (Matt, currently using PrEP, multiple partners)

**Figure 1:**
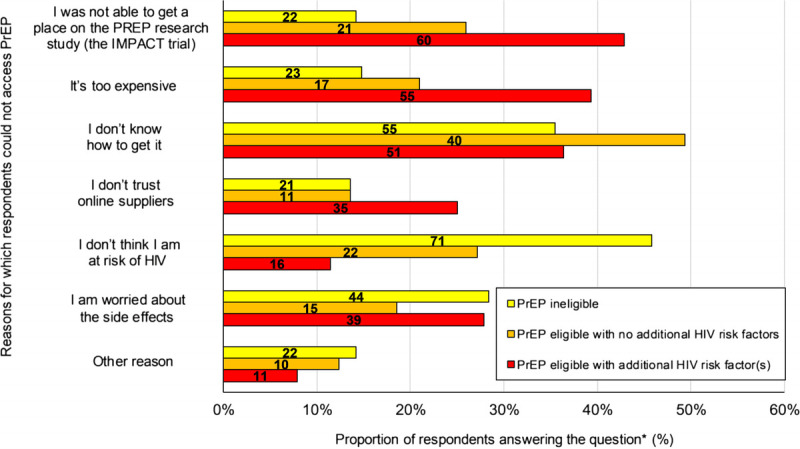
Reasons for not taking PrEP among MSM based on PrEP eligibility and HIV related risk factors. *Participants were deemed eligible for PrEP if reporting CAI in the past 3 months. The PrEP eligible population were subdivided into “elevated” and “lower” HIV risk groups based on the presence of 1 or more risk factors associated with greater odds of HIV infection. Self-identified risk factors (past 12 months) included: diagnosis of rectal chlamydia, rectal gonorrhea, or syphilis, use of PEP (postexposure prophylaxis), ≥5 partners or participation in chemsex (sexual intercourse under effects of mephedrone, crystal methamphetamine, or gamma hydroxybutyrate/gamma butyrolactone).

Lack of perceived HIV risk was survey respondents' main reason for not taking PrEP (Fig. 1). Interviewees spoke of choosing to take PrEP after assessing risks, benefits, and options (including access and dosing) based on their perceived HIV risk. Interviewees anticipated future PrEP use if their HIV risk increased, and 99/376 (26%) survey respondents anticipated using PrEP in the next 6 months.

“If the epidemiologists and health economists have sat down and said, “Yeah, you should consider—you have been chosen. You can help stop HIV!” then yes [I would take it]” (Liam, never used PrEP)

“I don't think I meet up with enough sexual partners to worry about having to use it [PrEP] at the moment but, in the future, if I have more partners then I would” (George, never used PrEP, in a relationship)

  Most survey respondents (and interviewees) who had never used PrEP would take it if it was free of charge (256/376, 68%), with higher proportions among those who were PrEP eligible or with elevated HIV-risk (171/221, 77%; 117/140, 84%). Those with elevated risk of HIV acquisition reported not being able to get a place on the Impact trial, cost and not knowing how to get PrEP as common barriers to PrEP use (Fig. 1).

PrEP side effects were not a concern for most survey respondents (Fig. 1) or interviewees due to regular check-ups and not experiencing adverse effects (themselves or others). Some were concerned about potential long-term effects and how to buy PrEP safely online.

“I wouldn't know where you would find it online because I'm a bit worried about finding things online” (Jim, never used PrEP, recently single)

### Impact of PrEP on Sexual Decision Making

The majority of HIV-negative/unknown status (473/578; 82%) and HIV-positive (31/39; 80%) survey respondents said PrEP use (themselves/partner) would reduce HIV transmission anxiety. Three hundred and thirty-nine (59%) of 578 HIV-negative/status unsure and 29 (74%) of 39 HIV-positive survey respondents would be more likely to have CAI with someone who was on PrEP than someone who was not on PrEP.

For some interviewees, potential higher risk of STIs was a reason given for not having sex with PrEP users, whereas conversely, other interviewees viewed PrEP users as more responsible and proactive regarding sexual health.

“When you see that someone's on PrEP you can probably think to yourself “this person's actually thinking about his sexual health. I could probably sleep with him” (Luke, currently using PrEP, casual partners)

**Figure 2:**
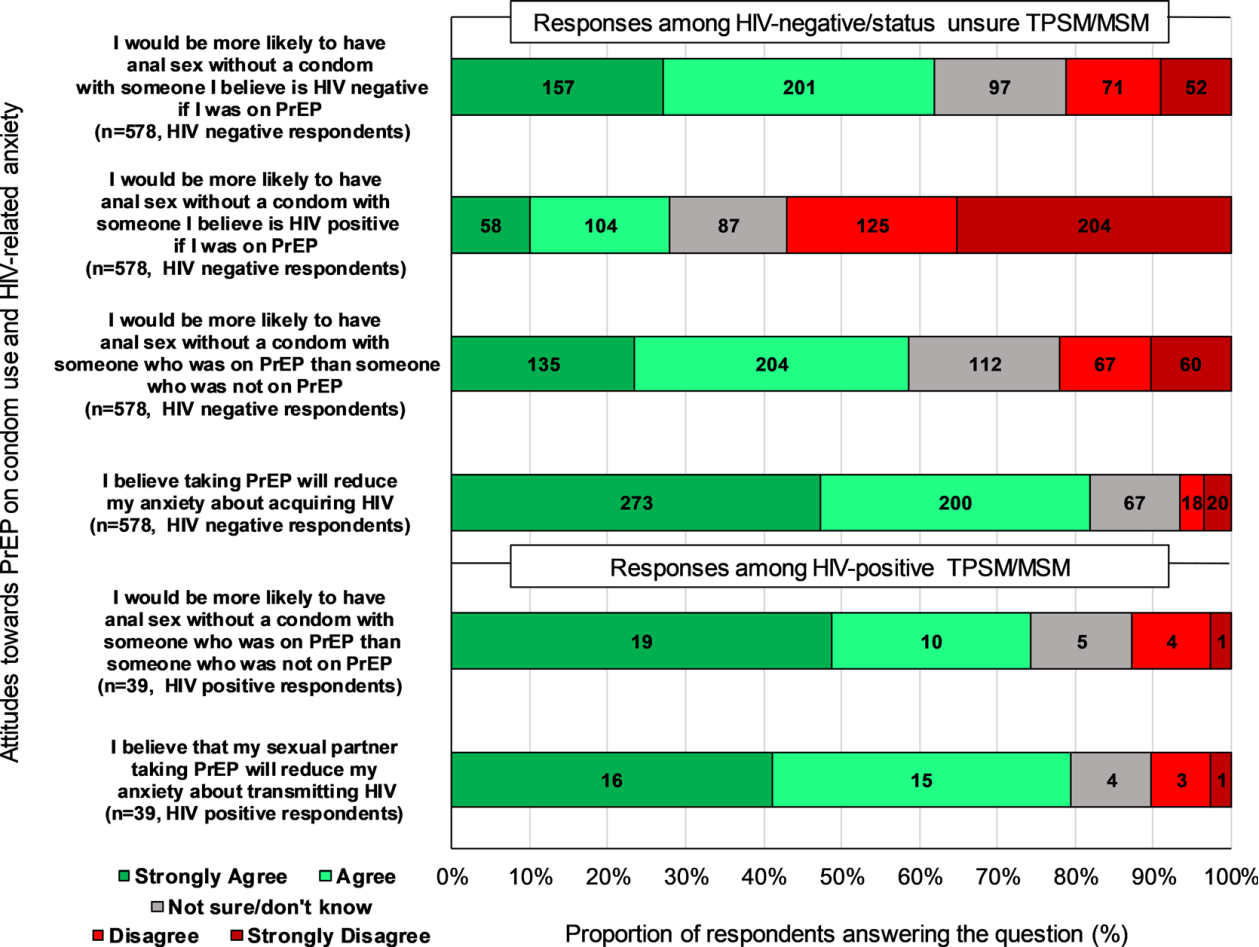
Predicted impact of PrEP on sexual risk taking.

  Of HIV-negative/status unsure survey respondents, 358 (62%) of 578 were more likely to not use a condom with someone they thought to be HIV-negative if they themselves were on PrEP (agree or strongly agree), whereas only 162 (28%) of 578 were more likely to do so with someone they thought to be HIV-positive (Fig. 2 and Supplemental Digital Content 2, http://links.lww.com/OLQ/A626).

Rather than never using condoms, interview data suggest that PrEP led to CAI only in certain situations, for example, with trusted partners, when drunk or when first on PrEP. A minority of interviewees used/would still use condoms with PrEP—because of STIs and to maximize HIV prevention.

“After I started [PrEP] I then thought “hold on, maybe now I can have more unprotected sex” which was perhaps foolish, but like I said before, the pendulum is swinging back the other way again. The initial excitement has waned” (Richard, currently using PrEP)

Interviewees fitted PrEP into a jigsaw of risk reduction strategies and some used it only for periods when they perceived their risk was higher.

“It's [PrEP] an extra layer of protection. That's how I see it. It's not protection, full stop, it's just an extra safety net if you like, so using that analogy, having a safety net, it's going to make me more likely to balance on the wire above.” (Kieran, currently using PrEP, a few regular partners)

### Risk Perception

To reduce risk of STIs, for some interviewees partner choice was based on perception of partner's STI status and the level of trust, with regularity of partners increasing level of trust. Some participants reported vetting processes to assess the risk of potential partners.

“I've got, like, regulars so no randoms coming around and that's it and I would never see them again. It's kind of like the same kind of people, which I think is a bit, I don't know, safer?” (Sam, currently using PrEP)

“I like to build up a level of confidence with—say if it's a one-off partner, a level of confidence through chatting and stuff before I'd meet them and I'd like to build up a level of trust, that they're being honest when they say they haven't got STIs” (Ross, never used PrEP)

  Many interviewees perceived the risk of STIs as unavoidable for MSM due to high prevalence and accepted that “STIs come with the territory” of gay sex. For some this inevitability of contracting STIs justified not changing their behavior to reduce the risk.

“I can't be obsessed about it [the STI risk] because it's just how it is. There's nothing really I can do” (Sam, currently using PrEP)

Some PrEP-using interviewees reduced the precautions they took when using PrEP e.g. engaged in more CAI, due to reduced concern about STIs.

“Because HIV is the one, is the one thing that isn't curable, or one of them that isn't curable, whereas the others [STIs] you can take a course of antibiotics, so I guess before I was taking it that's the one thing I was worried about whereas now I'm on PrEP it's a hugely reduced risk, so I [use condoms less]” (Simon, currently using PrEP)

  Concern about STIs was minimized by optimism that STI can be easily treated and are “curable,” compared with HIV.

“You can just get treated for whatever, all the STIs or whatever, so I guess I'm just a bit careless” (Luke, currently using PrEP, not in a relationship)

  There was a mixed level of knowledge and understanding about AMR and STIs. Most interviewees had heard of “super gonorrhea” and a minority understood that it meant being antibiotic resistant and difficult to treat. Many participants were concerned about antibiotic resistant STIs, described as “worrying” and “scary.” However, some participants appeared to try to invalidate concern about the risk of antibiotic resistance—saying they would only be concerned/change their behavior in the future if/when STIs become untreatable, or when it was in the news, or that it does not affect them personally.

“I'd like to think we hopefully won't get to the stage where oral sex is a complete no no, because you might be spreading super gonorrhoea around the place, that will never be able to be cleared, but if the prevalence [of super gonorrhoea] were to increase, maybe it would be time to—5 years down the track—to reassess that sort of perspective” (Liam, never used PrEP)

“The majority of the time I would have unprotected sex, um (pause) and I'd say specifically STIs aren't really what would change my mind about that, unless there was a change where a lot of people were not being able to be treated. I guess that would change things but at the minute it does not really impact on that decision” (Tom, currently using PrEP, in a relationship)

“If it was publicized that it's [super gonorrhea] becoming more common and harder to treat, I'd probably be a bit more cautious” (Ross, never used PrEP)

Despite the concern about antibiotic resistance, very few participants described modifying their behavior to reduce the risk of STIs. Many expressed guilt around this, saying it was “stupid thinking” and they “should” change behavior, but for some they did not want to think about it.

“No [change in risk behavior] because I think even though it's resistant it's still treatable so I sort of have this sense that we are not at the point yet where resistance is the same as untreatable” (Oliver, currently using PrEP, in an open relationship)

“I do not want to think about it too much because otherwise, I do not know, I just enjoy having sex and everything and I do not want to stop having that, um. But (sighs) I do not think I'm scared scared right now” (Sam, currently using PrEP)

For some, the risk of antibiotic resistance reinforced the justification for continuing to use condoms despite being on PrEP.

“If that news [super gonorrhea] had not cropped up, then I may have, sort of, moved towards thinking, “okay yeah, I know there is a risk there of STIs but that's okay, they are curable. I can get injections or medicine or whatever to get rid of it” and I'm on PrEP, therefore I'm protected from HIV, stick the condoms in a drawer and forget about them. Because of things like super gonorrhea and the risks that other infections may bring, I choose generally to keep using condoms to protect myself against that” (Kieran, currently using PrEP, a few regular partners)

“I'm [not] going to start going round and having loads of condom-less sex, because that's just not going to be the way that I will approach things because I'd be frightened of getting super gonorrhea and things” (Paul, previously used PrEP)

## DISCUSSION

The MSM/TPSM attendees were very enthusiastic about “life-changing” PrEP and its potential to reduce HIV prevalence and stigma. Knowledge about PrEP was common, and PrEP decision making was based on weighing up risks and benefits. However, barriers to PrEP use, particularly for those with elevated risk of HIV acquisition, were as follows: not knowing how to access PrEP, affordability, not being able to participate in the national “Impact” trial to access it for free.

Condomless anal intercourse was common among PrEP users because PrEP provides reduced HIV transmission anxiety. However, rather than always replacing condoms, PrEP was used flexibly in periods of increased risk.

For some participants, STIs other than HIV seemed inevitable/unavoidable and STI concerns were minimized by optimism that they are easily treated. Although there was widespread awareness of STI AMR, this was not seen universally as a current, personal risk necessitating changing sexual risk behaviors. However, many would change behavior if treatment resistant STIs were more widespread and/ or more widely publicized. Although, for some, the threat of antibiotic resistant STIs justified condoms use while being on PrEP.

The PrEP use in our clinic and the proportions of attendees using PrEP via the Impact trial are similar to other UK 2019 surveys.^7,21^ The enthusiasm for PrEP reflects Australian and American findings.^22^ The PrEP also had a significant impact on HIV transmission anxiety—improvements in emotional well-being and empowerment, as well as sexual satisfaction, intimacy, and liberation, which have been found previously.^13,23,24^ Not using PrEP because of a lack of access or knowledge appears similar for our sample of service users as an online survey of general MSM.^7^

Our study confirms that PrEP is used as one risk reduction tool of many, as an extra precaution.^13^ The finding that some HIV-negative MSM view those individuals taking PrEP as safer and therefore are more likely to engage in CAI with them is novel. Ambivalence about STIs (other than HIV) in decisions regarding CAI may reflect the ranking of certain STIs as “less scary” because of the familiarity with the treatment.^25^ Men-who-have-sex-with-men have been shown to be less concerned by the risks of bacterial STIs than those of HIV and hepatitis B and C.^25^ A systematic review^26^ suggests that PrEP's role in reduction of HIV incidence outweighs the potential side effects, a conclusion reached by many of our interviewees.

The high level of knowledge and informed decision making regarding PrEP may be due to the high-HIV-risk levels of our sample.^24,27^ In New York, MSM described PrEP-taking as leading them to reconsider their decision making about condom use and clarify their risk limits, irrespective of behavior change.^28^ The stigma of PrEP use (ie, presuming PrEP users are at a higher risk of STI due to perceived higher numbers of sexual partners) was less important than previous studies^13,24^—perhaps because of increasing awareness and use of PrEP in MSM communities^29^

When this study was undertaken, PrEP was not available in England on the NHS, and the number of individuals who could access PrEP free of charge was limited to those enrolled on the Impact trial. We found cost and lack of NHS-provision were key barriers to PrEP use. Given the enthusiasm for PrEP and that many men would use it if it was available at no cost, we anticipate a substantial increase in PrEP use when PrEP is made freely available in England. However, the COVID-19 pandemic disrupted sexual health services and access to PrEP, with the switch to telemedicine potentially disrupting continuity of PrEP provision, particularly for vulnerable groups.^30s^

The relationship between PrEP use and CAI/STI rates is complex. The increase in CAI and STI risk due to PrEP may be more nuanced and complex than a direct link.^13,16^^,31s^ As bacterial STI diagnoses rise, more infections will be treated, but potentially increasing the likelihood that untreatable resistant gonorrhea will circulate in the population and reduce the effectiveness of antibiotics over time. This is particularly pertinent for gonorrhea currently.^17^ Town et al recently concluded that public health actions to limit dissemination of AMR in England should aim to reduce risk behaviors that support *N. gonorrhoeae* transmission.^17^ Our study found that although high-risk MSM/TPSM were aware of bacterial AMR, the threat was perceived to be too distant to impact on their current behavior. Developing increased awareness of how infectious diseases are transmitted and prevented (because of the COVID 19 pandemic) could facilitate better understanding of STI AMR.^32s^

The PrEP is an additional tool in MSM's dynamic toolkit of HIV prevention strategies.^33s^ Health policy and practice should continue to focus on PrEP as an essential part of HIV combination prevention emphasizing the benefits, not only reducing the number of new HIV infections^33s^ but also in terms of reduced fear, improved emotional well-being and empowerment of PrEP users.

Information about STIs and AMR should be included in general risk reduction discussions, thus enabling MSM to understand the benefits of adopting strategies aimed at reducing the risk of bacterial STI AMR. These include; condom use, regular testing, completing treatment regimens, facilitating partner notification and, when appropriate, determining antimicrobial sensitivities and having a test of cure following treatment.^15^^,34s^ Clinicians and policymakers need to address this complexity in patient care, developing strategies to minimize the risks and improve the sexual health of patients.^23^ The PrEP may enhance HIV/STI screening among high-risk MSM who might otherwise not access these services.^23^

Improved education for MSM/TPSM on PrEP and AMR in STIs may lead to more nuanced ways of using and not using condoms. This requires further exploration in future studies codesigned with service users to best explore this complexity.

We had a 58% response rate from the clinic survey and retained an analytic sample of 31%, which is a limitation. However, this study achieved eligible responses representative of overall clinic attendees, although survey respondents were more ethnically diverse than the typical clinical population. The observational study design captured both NHS and private PrEP users, looking at PrEP access and attitudes in a time of limited NHS PrEP availability, highlighting service development and delivery issues at an individual and population level.

As sexual health clinic attendees, our sample was engaged with services and most were regularly testing. Groups not accessing sexual health services warrant further exploration. Most respondents were cis-male—future research should explore the views of TPSM and nonbinary individuals.

Integrating mixed methods data analysis allowed us to examine in depth the complexities of PrEP, reduced condom use, and risk perceptions. There is a need to understand how services can be tailored for those most at risk of HIV to attract into the service those who might benefit most.

The MSM/TPSM attendees at a UK NHS urban sexual health clinic were aware of and enthusiastic about PrEP. However, lack of Impact trial places at that time and cost of private purchase limited PrEP access. The PrEP may lead to increased CAI, but interviewees had good understanding of HIV acquisition risk and used PrEP as one risk reduction tool of many, as an extra precaution.

The reduction in anxiety about HIV transmission was striking, both for those who were HIV negative/unknown status acquiring the infection and for those who were HIV positive in terms of transmission.

The challenge remains to improve individual and national public health messaging with the aim of encouraging all those at risk of HIV acquisition to use PrEP but not to forget about other preventable STIs which are already becoming more difficult to treat at the population level. This is challenging when the consequences for the individual may seem remote, in the future and unrelated to their own sexual health. The impact of AMR and wider benefits of regular STI testing and judicious use of antibiotics may be easier to explain in the light of the health messaging during the recent COVID-19 pandemic.

## Supplementary Material

SUPPLEMENTARY MATERIAL

## Appendix

For further references, please see “Supplemental References,” http://links.lww.com/OLQ/A627.
