# Supplementary material for: Human Immunodeficiency Virus Preexposure Prophylaxis Knowledge, Attitudes and Perceptions of Sexual Health Risk in an Age of Sexually Transmitted Infection Antimicrobial Resistance
Source: Sex Transm Dis. 2021 Feb 3;48(9):685–92. doi: 10.1097/OLQ.0000000000001384 (PMC8360657; doi:10.1097/OLQ.0000000000001384)
Supplement: SUPPLEMENTARY MATERIAL [file std-48-0685-s001.pdf]

Supplemental Digital Content 1: interview topic guide

CHOP PrEP study
Service users interview topic guide (updated)

1. Introduction

Explain purpose of CHOP PrEP study. Objectives:

1. To examine views and experiences of at risk TPSM and MSM attending clinic and motivation for attending
2. To explore reasons for any change in sexual health seeking or risk taking behaviour
3. To investigate acceptability of different models of service delivery for testing to diagnose bacterial STIs and PrEP delivery for this client group

We are interested in your views and experiences of sexual health services and managing sexual health risk including what you think of PrEP.

I am going to start by asking you questions about your experience of attending Unity and things that influence how often you have sexual health check-ups. Then I will ask you about how you manage the risk of STIs and HIV separately. I'm going to ask you about these things separately because you may feel differently about STIs compared to HIV. I will ask you about this in relation to different types of sex – anal, oral sex and condom use. The last set of questions will be about PrEP.

Face to face: Discuss and sign consent form

Telephone interview consent:

- Introduction/aims/consent – voluntary participation, audio record, anonymous quotes
- Interview lasts 30-60 minutes is that OK?
- Any questions?
- Switch audio recorder on - For the audio recording, can I check that:
    - You read and understood the study information sheet?
    - You know that taking part in the interview is voluntary and you are free to stop the interview at any point and you may skip questions you would prefer not to answer?
    - You agree to our conversation being audio recorded?
    - You understand that quotations from the interview may be used to illustrate our findings but it will not be possible to trace who said them?
    - You understand that the information collected may be used to support other research in the future, and may be shared anonymously with other researchers?
    - You understand that authorised and responsible individuals may look at sections of the study data, to carry out monitoring for the research sponsor?
    - You agree to take part in the study?

2. Reasons/motivation for attending clinic

I'd like to start by discussing your reasons for recently attending Unity Sexual Health (Central Health Clinic) – thinking about the time you completed the questionnaire or your most recent visit and how you choose between the different services available.

- Have you been to Unity sexual health clinic (Central Health Clinic) before?

- What motivated you to attend Unity this time (e.g. worried about symptoms, recently been at risk, check-up, change in your sexual behaviour, new partner, advised to attend by someone else e.g. contact)?

- Why did you choose to come to the clinic to be tested over other methods? e.g. going to your GP or online/postal testing? (e.g. other methods not known, convenience, speed of results, anonymity)?

- Have you ever used online/postal testing kits?

3. Frequency of testing

Next, I'd like to talk about how frequently you go for sexual health testing and what things influence this decision.

- How regularly do you normally have a sexual health test?

- What things influence how regularly you test? (when more at risk of sexual infections, change of relationship status, signs and symptoms, recommendations?)

- *For MSM it is recommended to be tested every three months.* What do you think about this recommendation? If they already mentioned the recommendation, ask where they heard of it.
    - Is it possible for you to test every three months?
    - What would prevent you testing every three months (e.g. service design – access to appointments etc.)?

- What does/would help you to test every 3 months (e.g. service design – access to appointments etc.)?
    - online/postal testing?
    - Are there any situations where you would not want online/postal testing? (e.g worried about symptoms, recently been at risk)

Unity are now using a new rapid results service as part of regular SH screening. Results are available in about 4 hours for chlamydia and gonorrhoea (HIV tests are also done but still takes longer – up to 3 weeks - to get the results). –

- Did you have this service?

In this rapid results service - Patients either receive a text message notifying them of negative results or if results are positive they are asked to return to the clinic that afternoon or the next day

to receive appropriate treatment. This means treatment can be based on their test results. Previously, if patients had symptoms or were suspected to be positive, they would receive treatment immediately as a precautionary measure, as they could wait over a week for the test results.

- How acceptable would/did you find waiting for 4 hours/until the next day to receive treatment for chlamydia and gonorrhoea only after your test results are known (instead of being given treatment straight away 'just in case')?
- Would having a rapid results service encourage you to test more regularly? Why/why not?
- Are there any situations you would prefer not to have this rapid results service?

4. STI Risk

I now want to ask about sexually transmitted infections (STI) other than HIV.

- Could you tell me a bit about your relationship status and what kind of sexual partners you tend to have at the moment? Do you have: long-term partner, open/exclusive relationship, regular casual partners, one night stands/hook ups
    - [if not in a monogamous relationship] how do you most often meet sexual partners e.g. through friends, gay scene, dating/hook up apps e.g. Grindr, social networking sites e.g. facebook etc

- Does the risk of STIs other than HIV impact on your sex life?
    - Who you have sex with?
    - How you have sex? Anal sex? Oral sex? Bottom or top position? Condoms?

- How do you manage your risk of STIs with sexual partners (do you have conversations about STI status, condom use)?
    - Does this differ for casual vs regular vs long-term (e.g. husband) partners? How?
    - If currently in a monogamous relationship - did you manage your risk of STIs differently before you were in a relationship (over the last year)?

- Have you heard of antibiotic-resistant or "super" Gonorrhoea? If yes – what have you heard about it (what it is/the implications/what causes it)? If no - *Explanation: There have been a handful of cases of super Gonorrhoea, which is caused by the over use of antibiotics. As a result, antibiotics that used to work in the past to treat Gonorrhoea are less effective.*

- Has the risk of super Gonorrhoea changed how you have sex? (condoms) Might it change it in the future?

5. Risk management HIV

I now want to talk about how people manage the risk of HIV.

- Does the risk of HIV impact on your sex life (e.g. the type of sex you have – anal, oral, bottom or top, condom use)?

- Does risk of HIV influence who you choose to have sex with?

- How do you negotiate sex with a partner in relation to HIV (knowledge/talk about HIV status, PrEP)?
    - Does this differ for casual vs regular partners? How?

- How do you manage your risk of getting HIV in terms of your sexual behaviour (type of sex, regular condoms, PEPSE, PrEP, etc.)?

- Do you use dating apps to find partners? (e.g. Grindr, scruff)

- What profile information influences who you decide who to have sex with?

- What is your attitude towards having sex with people who are HIV positive?

- Have you heard of U=U or undetectable viral load?

    - If yes what do you know about it? What is your attitude towards having sex with people who are HIV positive on treatment and have an undetectable viral load? Examples?
    - If no *U=U is when a person is living with HIV is on effective treatment, which lowers the level of HIV in the blood, referred to as an undetectable viral load. At this stage, HIV cannot be passed on sexually. U=U means "Undetectable = Untransmittable.*
      What is your attitude towards having sex with people who are HIV positive? What if they are on treatment and have an undetectable viral load?

6. PrEP use

The last set of questions are about PrEP.

- Have you heard of PrEP before? If so, how? What do you know about it? (how it works, what it is for)

PrEP is a medicine for HIV negative people, is taken before and after sex and can reduce the risk of acquiring HIV when taken as instructed. At the moment, PrEP is not available on the NHS in England, but NHS England is currently conducting the PrEP Impact trial.

- What do you think of PrEP as a way of reducing risk of getting HIV?
  - Does anything concern you about PrEP?
  - Have you ever taken PrEP? Are you currently taking PrEP?
  - Have you discussed PrEP with a doctor (including GP)?

*[If **yes to PrEP use**]*

- What motivated you to use PrEP?

- Where did you get PrEP (as part of a research study, doctor, PEP/PEPSE you acquired and split, Internet, Friend)?
  - Have you faced any barriers getting PreP? (not able to get a place on a PrEP research study (e.g. Impact Trial), too expensive, know/don't know how to get it, trust/don't trust online suppliers)

- How often are you taking PrEP (daily, event based)?
  - What things influence how often you take PrEP?
  - Do you experience any issues taking PrEP as instructed? If so, what are these issues?
  - How did you decide how often to take PrEP (event-based etc) (what information etc). Do you feel you need any more information on this?

- Have you told people you are taking PrEP? How have friends/healthcare professionals reacted? How have sexual partners reacted? (stigma related to PrEP use)
  - Why do you think people react this way? Have you noticed certain people or groups react in certain ways?

- Have you noticed any changes generally in the MSM community since PrEP was introduced?
- How would you describe the impact of taking PrEP on your life, including your sex life, compared to before you started using PrEP?
  - Influenced who you have sex with? (who have sex with, number of sexual partners)
  - Influenced sexual behaviour, how you have sex? (e.g. reduced anxiety about acquiring HIV, enhances sexual satisfaction, risk taking, condom use)
  - Influenced your relationships with sexual partners (intimacy, trust, communication about STIs/HIV)
  - How often you have a sexual health check?
  - Has PrEP has any impact on the rest of your life (not sex related) e.g. mental health/anxiety, empowerment, your other relationships? What are the reasons for these impact?

- Do you have any concerns about using PrEP? (don't trust online suppliers, worried about how effective it is/ worried about side effects)
- Have you noticed any changes generally in the MSM community since PrEP was introduced?

- Is there any more information you want to know about PrEP?

- What do you now know about PrEP that you wished you knew before taking it?

- What do you think are the best ways to spread the word about PrEP?

- Do you think PrEP should be available on the NHS? Why/why not?

*[If **no to PrEP use**]*

- Who do you think PrEP is for? What sort of person takes PrEP? (stigma related to PrEP use)

- How would/do you react if a friend told you they are taking PrEP? (stigma related to PrEP use)

- How would/do you react if a prospective sexual partner told you they are taking PrEP? (stigma related to PrEP use, condom use). What do you think this reaction is based on?
    - Are you more or less likely to have sex with someone who uses PrEP?
    - If someone said they were taking PrEP, would it change the type of sex you have? (condoms)

  - Have you noticed any changes generally in the MSM community since PrEP was introduced?
- Would you be interested in taking PrEP? Why / why not (able/not able to get a place on a PrEP research study, too expensive, know/don't know how to get it, trust/don't trust online suppliers, think/don't think at risk of HIV, worried/not worried about side effects, worried about how effective it is, stigma related to PrEP use)? Are there certain times or situations you might consider taking PrEP?

- If you were to use PrEP, do you think it would have an impact on your sexual life/sexual behaviours? (e.g. risk-taking including condom use, reduced anxiety about acquiring HIV, enhances sexual satisfaction, changes in who have sex with, number of sexual partners) How?

- What information would you want to know before taking PrEP? How would you like to receive this information?

- What do you think are the best ways to spread the word about PrEP?

  - Do you think PrEP should be available on the NHS? Why/why not?

7. Close

Before we finish is there anything I haven't covered today that you would like to add?
End of audio recording / thanks for participating. **Provide debrief sheet.**

Ask for address if telephone interview.
