# Supplementary material for: Human Immunodeficiency Virus Preexposure Prophylaxis Knowledge, Attitudes and Perceptions of Sexual Health Risk in an Age of Sexually Transmitted Infection Antimicrobial Resistance
Source: Sex Transm Dis. 2021 Feb 3;48(9):685–92. doi: 10.1097/OLQ.0000000000001384 (PMC8360657; doi:10.1097/OLQ.0000000000001384)
Supplement: SUPPLEMENTARY MATERIAL [file std-48-0685-s002.pdf]

Supplemental Digital Content 2: Supplementary figures

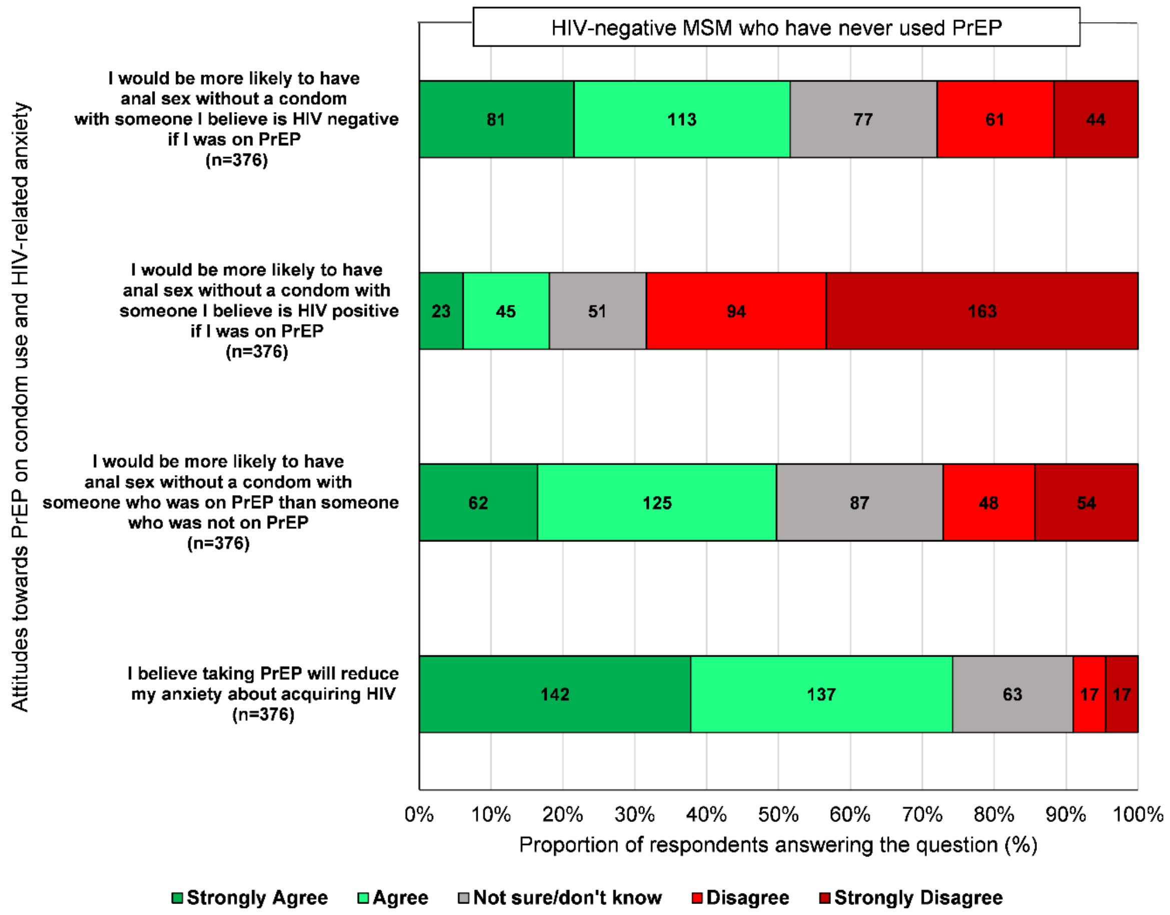

Figure S1. Proportion of HIV-negative TPSM/MSM/TPSM who have never used PrEP from the survey data who indicated agreement or non-agreement with statements concerning PrEP use and likelihood to use or forgo condoms.

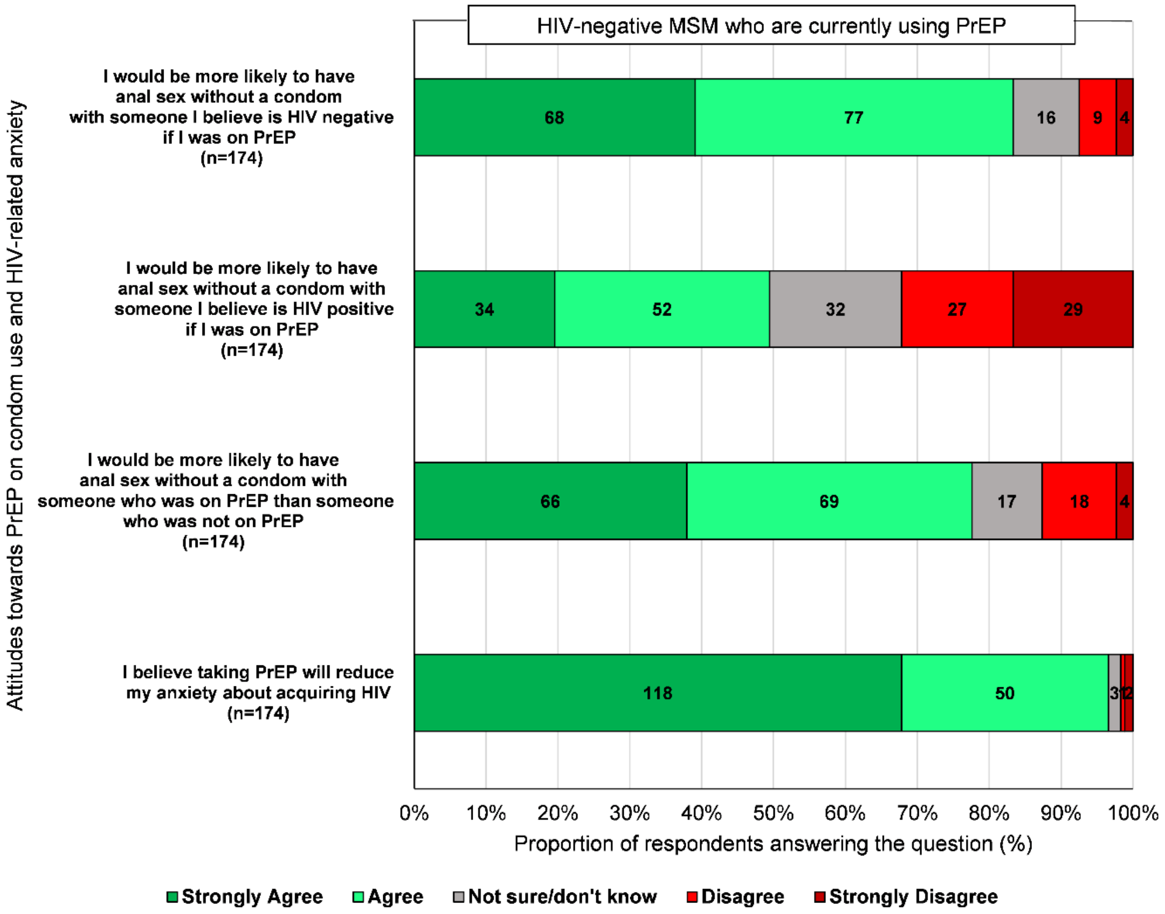

Figure S2. Proportion of HIV-negative TPSM/MSM/TPSM who are currently using PrEP from the survey data who indicated agreement or non-agreement with statements concerning PrEP use and likelihood to use or forgo condoms.

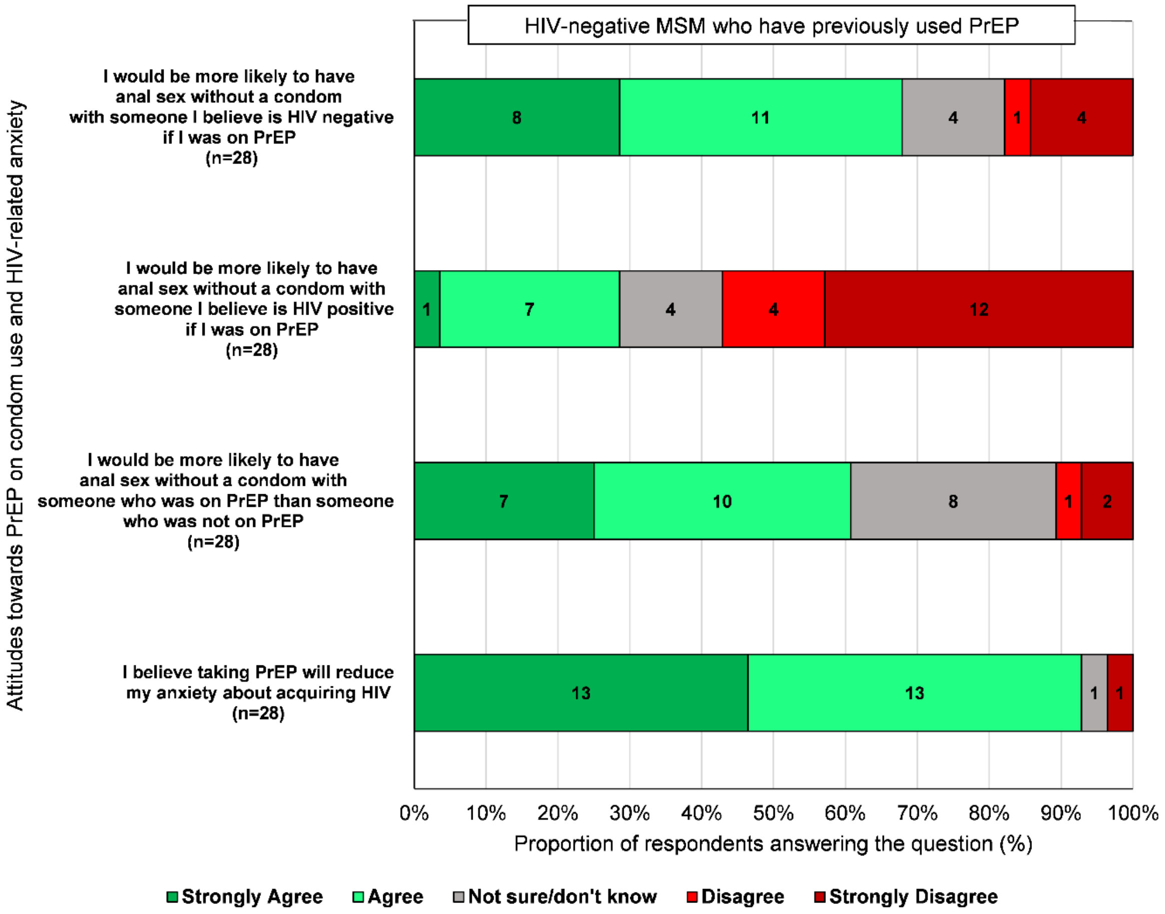

Figure S3. Proportion of HIV-negative TPSM/MSM/TPSM who have previously used PrEP from the survey data who indicated agreement or non-agreement with statements concerning PrEP use and likelihood to use or forgo condoms.
