# Supplementary material for: Human Immunodeficiency Virus Preexposure Prophylaxis Knowledge, Attitudes and Perceptions of Sexual Health Risk in an Age of Sexually Transmitted Infection Antimicrobial Resistance
Source: Sex Transm Dis. 2021 Feb 3;48(9):685–92. doi: 10.1097/OLQ.0000000000001384 (PMC8360657; doi:10.1097/OLQ.0000000000001384)
Supplement: SUPPLEMENTARY MATERIAL [file std-48-0685-s003.pdf]

31. BASSH. BASHH COVID-19 SURVEY FINDS OVER HALF OF SERVICES HAVE BEEN CLOSED, 2020.

32. Traeger MW, Cornelisse VJ, Asselin J, et al. Association of HIV Preexposure Prophylaxis With Incidence of Sexually Transmitted Infections Among Individuals at High Risk of HIV Infection. *JAMA* 2019;321(14):1380-90.

33. Town K, Harris S, Sanchez-Buso L, et al. Genomic and Phenotypic Variability in Neisseria gonorrhoeae Antimicrobial Susceptibility, England. *Emerg Infect Dis* 2020;26(3):505-15.

34. Gafos M, Horne R, Nutland W, et al. The Context of Sexual Risk Behaviour Among Men Who Have Sex with Men Seeking PrEP, and the Impact of PrEP on Sexual Behaviour. *AIDS Behav* 2019;23(7):1708–20.

35. Hillis A, Germain J, Hope V, et al. Pre-exposure Prophylaxis (PrEP) for HIV Prevention Among Men Who Have Sex with Men (MSM): A Scoping Review on PrEP Service Delivery and Programming. *AIDS Behav* 2020;24(11):3056–3070
